# Novel rabies vaccine candidates development based on pseudotyped lentiviral vectors with rabies virus glycoprotein

**DOI:** 10.1371/journal.pntd.0013404

**Published:** 2025-08-26

**Authors:** Lifang Shuai, Mingyu Xu, Nana Pei, Cheng Zhu, Marwa Belghait, Zhiming Hu, Jinlong Li, Hongwei Li, Yingying Mao, Hongyan Du

**Affiliations:** 1 South China Institute of Biomedicine, Guangzhou, Guangdong, China; 2 School of Laboratory Medicine and Biotechnology, Southern Medical University, Guangzhou, Guangdong, China; 3 Department of Clinical Pathology, The First Affiliated Hospital of Jinan University, Guangzhou, Guangdong, China; The University of Kansas, UNITED STATES OF AMERICA

## Abstract

Rabies, caused by the rabies virus (RABV), remains a global public health issue. Traditional inactivated rabies vaccines are costly, risky, and require multiple doses for post-exposure prophylaxis. The rabies virus glycoprotein (RABV-G), essential for inducing protective antibodies, is crucial for new vaccine development. Lentiviral vectors offer promise due to their efficient gene delivery and strong immune responses. We designed three recombinant pseudotyped lentiviral vector vaccines with enhanced green fluorescent protein (eGFP) as marker, among VSV-G/LV-RABV-G the RABV-G only lies in the core of pseudotyped lentiviral particle, among RABV-G/LV-RABV-G the RABV-G lies in both of the core and the envelop and among RABV-G/LV-eGFP the RABV-G only lies in the envelop. These were tested for antigenicity, infectivity, and neutralizing antibody response. All vaccines showed strong antigen specificity and high titers for virus particles production. Immunization tests in mice showed that VSV-G/LV-RABV-G and RABV-G/LV-RABV-G vaccines induced high neutralizing antibody levels within 3 days, sustained up to 10 weeks. The RABV-G/LV-eGFP vaccine, especially with CPG-ODN adjuvant, also generated significant antibody responses. In summary, the recombinant pseudotyped lentiviral vector vaccines based on the RABV-G show promise for effective, single-dose rabies vaccination.

## Author summary

Rabies remains a serious public health problem in Asia and Africa, which covers a population of over 3 billion in endemic areas. According to the World Health Organization's estimation, approximately 59,000 people die from rabies worldwide each year. Rabies is an infectious zoonotic disease caused by the Rabies virus (RABV), mainly transmitted by infected animals through saliva bites. Under normal circumstances, after a person is attacked and infected by an animal carrying the rabies virus, the incubation period may range from several days to several years. At present, rabies is mainly prevented and intervened through rabies vaccination before or after exposure. The rabies vaccines currently available on the market cannot provide 100% protection in post-exposure prophylaxis due to their varying quality and the long time it takes to produce neutralizing antibodies. Some rabies virus-infected individuals still experience the virus entering the central nervous system, leading to death. Consequently, the development of novel rabies vaccines characterized by reduced production costs, simplified immunization schedules, and enhanced immunogenicity remains a critical strategy for rabies prophylaxis and post-exposure management. This investigation proposes an innovative framework for creating new vaccine candidates that integrate these optimized characteristics.

## 1. Introduction

Rabies, caused by the rabies virus (RABV), continues to pose a significant threat to global public health, with an estimated 59,000 human deaths annually, primarily in Asia and Africa [1–3]. Traditional rabies vaccines, which are based on inactivated virus, have limitations including high production costs, the need for multiple doses, and potential risks associated with their administration [2,4]. Consequently, there is an urgent need for more effective, safer, and more accessible rabies vaccines.

The rabies virus glycoprotein (RABV-G) is the key antigen for inducing protective immunity against rabies. RABV-G is responsible for viral attachment and entry into host cells, making it a prime target for vaccine development [5–7]. Advances in genetic engineering and vector technology have paved the way for innovative vaccine platforms. The recombinant virus particles can achieve high titers through purification, and the production process is safe and convenient, which has garnered wide attention in the research and development of viral vector vaccines [8,9].

During the current development of vaccines with viral vector, lentiviral vectors have emerged as a promising candidate. The lentiviral vector system has significant potential in the development of new rabies vaccines due to its efficient gene delivery mechanism, biological safety, and ability to stimulate the immune system [10,11].

Currently, clinical trials have been conducted on HIV vaccines based on lentiviral vectors [12,13]. Early immune response and protection against rabies virus attacks are crucial, and lentiviral vector systems have been shown to induce an early and persistent humoral immune response to other viruses with a single injection [14,15]. Iglesias *et al*. constructed a lentiviral vector vaccine expressing the west nile virus glycoprotein, which produced complete challenge protection in mice within 3 days after a single injection. Studies have demonstrated the potential of lentiviral vectors in inducing early and sustained humoral immune responses [16]. Therefore, the development of new rabies vaccines based on the lentiviral vector system holds promising prospects.

In this study, we designed three novel RABV recombinant lentiviral vaccines including the green fluorescent protein (GFP) marker, which are VSV-G/LV-RABV-G, RABV-G/LV-RABV-G, and RABV-G/LV-eGFP. Their construct respectively utilized VSV-G and RABV-G for pseudotyping to optimize antigen delivery and presentation. The immune effects of the recombinant lentiviral vaccines were compared and the last one, RABV-G/LV-eGFP, was tested with and without the CpG oligodeoxynucleotide (CpG-ODN) adjuvant to evaluate its adjuvant effect using GFP for tracking purposes. Our results suggest that recombinant lentiviral vector vaccines incorporating RABV-G are promising candidates for effective, single-dose rabies vaccination, which lay the groundwork for further development and potential clinical application of these innovative rabies vaccine strategies.

## 2. Materials and methods

### Ethics statement

Written informed consent was obtained from each participant, and the study protocols were approved by the Ethics Committee of Nanfang Hospital. The animal experiment protocol was reviewed and approved by the Ethics Committee of Experimental Animals at Southern Medical University.

### 2.1 Cells culture and plasmids construction

Human embryonic kidney (HEK) 293T cells (ATCC, Manassas, VA, Catalogue #: CRL-3216) were cultured following the ATCC cell culture guide. The enhanced green fluorescent protein (eGFP) recombinant lentiviral expression plasmid, pLV-eGFP, containing cytomegalovirus (CMV) promoter, was constructed in our laboratory [17]. The packaging plasmids pCMV-VSV-G and psPAX2 were obtained from Dr. Junming Yue (Department of Pathology, University of Tennessee Health Science Center). The codon-optimized RABV-G gene sequence was designed by our research group and synthesized by Anhui General Biological System Co., LTD. The sequence of the cDNA fragment was derived from the rabies virus strain CTN-1V5G (Accession number: JN234418).

The two recombinant plasmids, pCMV-RABV-G and pLV-RABV-G, were constructed based on envelop plasmid, pCMV-VSV-G and transfer plasmid, pLV-eGFP. For the construction of pLV-RABV-G, the pLV-eGFP plasmid was digested with *Nhe I* and *Mlu I* restriction enzymes, and the RABV-G gene was inserted into pLV-eGFP to yield pLV-RABV-G (S1A Fig). The pCMV-RABV-G plasmid was constructed by substituting VSV-G fragment in Lentivirus envelope plasmid pCMV-VSV-G with RABV-G (S1B Fig). Both recombinant plasmids were validated by restriction enzyme digestion and partial sequencing.

### 2.2 Production and characterization of pseudotyped recombinant lentivirus

**Fig 1:**
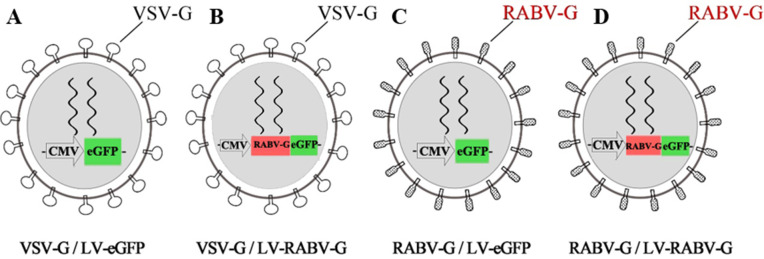
Schematic illustration and theoretical structure of pseudotyped lentiviral particles. **A**. Control pseudotyped lentiviral particles VSV-G/LV-eGFP, which was constructed with envelope plasmid pCMV-VSV-G, core plasmid pLV-eGFP and packaging plasmid psPAX2. **B**. Pseudotyped lentiviral particles VSV-G/LV-RABV-G, which was constructed with envelope plasmid pCMV-VSV-G, core plasmid pLV-RABV-G and packaging plasmid psPAX2. **C**. Pseudotyped lentiviral particles RABV-G/LV-eGF, which was constructed with envelope plasmid pCMV-RABV-G, core plasmid pLV-eGFP and packaging plasmid psPAX2. **D**. Pseudotyped lentiviral particles RABV-G/LV-RABV-G, which was constructed with envelope plasmid pCMV-RABV-G, core plasmid pLV-RABV-G and packaging plasmid psPAX2.

The blank plasmid, pLV-eGFP and the two recombinant plasmids, pCMV-RABV-G and pLV-RABV-G, with package plasmid pCMV-VSV-G and psPAX2 were assembled to three recombinant rabies lentiviral particle systems, VSV-G/LV-RABV-G, RABV-G/LV-eGFP, and RABV-G/LV-RAVB-G, and the one control, VSV-G/LV-eGFP. The biggest difference between them lies in whether RABV-G is placed in the virus core or the outer envelope. The schematic illustration and theoretical structure of pseudotyped lentiviral particles was shown in Fig 1. And the different plasmids prepared for producing the pseudotyped virus particles were as shown in table 1.

**Table 1:** The combined modes of plasmids for pseudotyped recombinant lentivirus production.

Pseudotyped recombinant lentivirus		Envelope plasmid	Transfer plasmid	Packaging plasmid
Control	VSV-G/LV-eGFP	pCMV-VSV-G	pLV-eGFP	psPAX2
recombinant lentivirus	VSV-G/LV-RABV-G	pCMV-VSV-G	pLV-RABV-G	psPAX2
	RABV-G/LV-eGFP	pCMV-RABV-G	pLV-eGFP	psPAX2
	RABV-G/LV-RABV-G	pCMV-RABV-G	pLV-RABV-G	psPAX2

The different plasmids were packaged to produce these lentiviral particles according to our previously established methods [18]. 5 × 10^6^ HEK 293T cells were seeded in a 10 cm dish and later transfected at 90% confluency. The different plasmids composition suspended in 500µL 2mM CaCl_2_ solution was then added dropwise, while vortexing, to an equal volume of 2 × HBS, for total of 1mL. This mixture was added to the 10 cm dish for transfection of HEK293T cells and maintained in 5% CO_2_ at 37°C. The packaged recombinant lentiviruses were harvested from the supernatant of the cell cultures at 48hrs post transfection and subsequently centrifuged at 4°C, 1520 × g for 10min to remove cell debris, followed by concentration using a 100K concentration column at 4°C, 9500 × g for 30min.

### 2.3 Purification of recombinant lentivirus with sucrose density gradient ultracentrifugation

The concentrated lentiviral vector was then transferred to an ultracentrifuge tube. 40% sucrose solution was carefully added to the bottom of the tube, followed by slow addition of a 60% sucrose solution beneath the 40% layer to create a density gradient. The tube was then centrifuged at 109,600 × g for 120 minutes at 4°C. Upon completion of centrifugation, the ultracentrifuge tube was carefully removed, and the white viral band located at the interface between the 40% and 60% sucrose layers was collected. This viral band was resuspended in an appropriate volume of PBS. The resuspended solution was then centrifuged at 80,000 × g for 90 minutes at 4°C to pellet the virus. Subsequently, the supernatant was discarded, and the viral pellet was resuspended in PBS to achieve purified recombinant lentivirus.

### 2.4 Quantification of pseudotyped recombinant lentivirus titer by qPCR

The titer of the purified recombinant lentivirus was determined using a lentivirus titer assay kit (Applied Biological Materials (ABM) Inc., Canada). Following the instructions, 10-fold serial dilutions of the Standard Control DNA were prepared by adding 5μL of DNA to 45μL of nuclease-free H₂O. Dilutions from 1/10–1/10,000 will be used for generating the standard curve. For purified high titer viral samples, dilute the virus to 10^6^-10^8^ IU/mL range (for best results) with PBS. The mixture including viral sample were detected by qPCR instrument (ABI7500, USA) under cycling condition provided from instruction. The recombinant lentivirus titer was calculated with Ct value according to the conversion formula in the lentivirus titer assay kit.

### 2.5Western blotting analysis of rabies glycoprotein expression

For detecting RABV in recombinant lentiviral vector expression or pseudotyped recombinant lentivirus in host cells.The cell lysates collected from HEK293T cells transduced with the vector or concentrated solution of pseudotyped recombinant lentivirus were mixed with SDS loading buffer and boiled for 10min. After centrifugation at 9500 × g for 5min, 20µL supernatant was loaded onto SDS-PAGE gels for electrophoresis. Proteins were then transferred to a PVDF membrane. The membrane was blocked with 10% skimmed milk powder at room temperature for 2hrs. Next, the membrane was incubated overnight at 4°C with human anti-RABV serum (1:500 dilution) obtained from a person vaccinated with human Vero-cell rabies vaccine, followed by washing with TBST five times. Subsequently, the membrane was incubated at room temperature for 1hr with HRP-labeled rabbit anti-human IgG secondary antibody (Beijing BioRadars biological technology CO.,LTD, diluted 1:20,000). After another five washes with TBST, a luminescent solution was added, and the results were visualized using a gel imaging system.

### 2.6 Mice immunization with pseudotyped recombinant lentiviruses

SPF-grade female BALB/c mice, aged 4–6weeks, were purchased from the experimental animal center in Southern Medical University. 48mice were randomly divided into 8 groups and injected at the quadriceps femoris of hind limb with 1 × 10^7^ transducing units (TU)/every mouse of pseudotyped recombinant lentivirus from different groups. The grouping and immunizing mode and dosage of mice are listed in Table 2. If secondary immunization is required, a secondary immunization is given 14days after the initial immunization. The immune response was monitored for up to 10weeks, during which blood samples were collected before immunization and at 3^th^ day, followed by weekly collections, via posterior orbital venous plexus puncture in mice.

**Table 2:** Grouping and immunity of BALB/c mice.

No.	Pseudotyped recombinant lentivirus	IMD(TU/mouse)	Adjuvant	Immune mode
1	VSV-G/LV-RABV-G	1 × 10^7^	—	i.m.
2	VSV-G/LV-RABV-G	1 × 10^7^	—	i.p.
3	RABV-G/LV-RABV-G	1 × 10^7^	—	i.m.
4	RABV-G/LV-eGFP	1 × 10^7^	—	i.m.
5	RABV-G/LV-eGFP	1 × 10^7^	CpG-ODN 1018	i.m.
6	RABV-G/LV-eGFP	1 × 10^7^	CpG-ODN 1018	i.m. Two-dose
7	VSV-G/LV-eGFP	1 × 10^7^	—	i.m.
8	PBS	1 × 10^7^	—	i.m.

Note: i.m. = intramuscular injection; i.p. = intraperitoneal injection

Adjuvant: CpG-ODN 1018 (sequence 5’-TGACTGTGAACGTTCGAGATGA-3’) synthesized by Shenggong Bioengineering (Shanghai) Co., LTD was diluted and dissolved in endotoxin-free water.

### 2.7 RABV neutralization test using a pseudotyped recombinant lentivirus with RABV-G envelop

A RABV neutralization test was operated using a pseudotyped recombinant lentivirus, RABV-G/LV-eGFP, with RABV-G as the envelope protein We referenced previously published methods [19,20] and made some adjustments ourselves. Initially, serum samples containing a known titer of rabies virus neutralizing antibodies (1.5 IU/mL) were heat-inactivated at 56°C for 30 minutes. Negative serum samples underwent the same treatment. Following heat inactivation, fresh DMEM medium was added for 2-fold gradient dilution starting from 1:2, with each dilution repeated across 5 wells, resulting in a final volume of 50μL of serum dilution in each well.

The recombinant lentivirus RABV-G/LV-EGFP was diluted to a concentration of 50TCID_50_/50μL. Then, 50μL of the diluted lentiviral vector was mixed with 50 μL of each serum dilution. This mixture was incubated at 37°C for 1hr. Subsequently, 100μL incubated mixture was added to HEK293T cells in a 96-well plate and cultured. After 72hrs, the cells were examined under a fluorescence microscope. Wells displaying fluorescence were designated as infected, while those lacking fluorescence were classified as neutralized. Calculate the neutralizing antibody titer for the test serum using the following formula: Neutralizing Antibody Titer (IU/mL) = 1.5×(At/Ac)×2^(Bt-Bc)/5^. At represents the highest serum dilution in the test serum wells with no fluorescent cells. Ac represents the highest serum dilution in the positive control serum wells with no fluorescent cells. Bt is the number of wells with no fluorescent cells at the At dilution of the test serum. Bc is the number of wells with no fluorescent cells at the Ac dilution of the standard serum. Neutralization Test was **r**epeated at least three times to obtain neutralizing antibody titers.To evaluate neutralizing antibody titers in unknown serum samples, both the recombinant rabies pseudovirus and the fluorescent antibody virus neutralization (FAVN) test [21,22] were employed. A correlation analysis was conducted to compare the results obtained from these methods for determining neutralizing antibody titers.

### 2.8 Indirect enzyme-linked immunosorbent assay (iELISA) analysis for serum samples

To assess the antigenic characteristics of RABV-G expressed from recombinant lentivirus vectors, an indirect ELISA was conducted. ELISA plates (Costar, Corning, US) were coated with 100μL carbonate-bicarbonate coating buffer (0.05M, pH9.6) containing either purified rRABV G (100ng/well) produced in-house in 293T cells and incubated overnight at 4°C. When evaluating the specific reaction of recombinant lentiviral particles with serum, after purified the culture supernatant from the viral packaging system was diluted with PBST(1:100) as the coating antigen. To minimize nonspecific binding, plates were blocked with 10% skim milk (100 μL/well). Serum samples, diluted 1:100 in PBS supplemented with 10% skim milk (BIO-RAD, USA), were added in triplicate (100μL/well) and incubated at 37°C for 1 hr. Plates were washed five times with PBST (PBS with 0.05% Tween-20) and subsequently incubated with HRP-conjugated goat anti-human antibodies (Abcam, USA) for 1 hr at 37°C. Following an additional five PBST washes, 100μL of tetramethylbenzidine substrate (TMB) solution (Genstar, Shanghai) was added to each well and incubated for 10min at 37°C. The reaction was halted by adding 50μL H_2_SO_4_ solution (2M) per well. Absorbance at 450nm was measured using a microplate reader (Bio-Rad, USA).

Human serum samples used in this study were obtained from individuals vaccinated with a commercial human Vero-cell Rabies vaccine, and their antibody levels against RABV were previously assessed using the FAVN test at the OIE Reference Laboratory for Rabies in Changchun, China. Sera from non-vaccinated individuals were collected from healthy volunteers at physical examination center in Nanfang Hospital (Guangzhou, Guangdong, China).

### Statistical analysis

Data from multiple experiments were presented as the mean±standard deviation (SD). Statistical analysis of all experimental data was performed using SPSS 20.0 software and Graphpad Prism 10.0 for Windows. Shapiro-Wilk test and visual inspection with Q-Q plots to evaluate normality were employed to evaluate data normality. Levene test were conducted for assessing data homogeneity of variance. When the data satisfied normality and homogeneity of variance, one-way ANOVA and two-way ANOVA were employed for comparison among multiple groups. Post hoc test by using Tukey HSD test were conducted to perform two fold comparisons between multiple groups. A significance level of **p* *< 0.05 was considered statistically significant.

## 3. Results

### 3.1 Production and characterization of pseudotyped recombinant lentivirus

**Fig 2:**
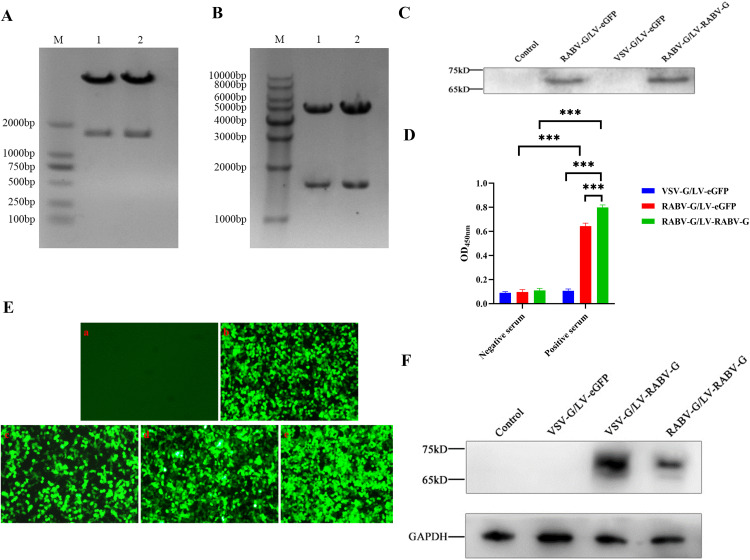
Construction and Characterization of pseudotyped lentiviral vectors/particles with rabies virus glycoprotein. **A.** Restrction enzyme digestion analysis of pLV-RABV-G. M: DL2000 DNA marker; 1 and 2 line: Double enzyme digestion product of pLV-RABV-G with *Nhe* I + *Mlu* I. **B.** Restrction enzyme digestion analysis of pCMV-RABV-G. M: 10000 bp DNA marker, 1 and 2 line: Double enzyme digestion product of pCMV-RABV-G with *Nhe* I and *Mlu* I. **C.** Analysis of Rabies virus RABV-G expression in recombinant pseudotyped lentiviral particles packaged by HEK293T cells by western blot. **D.** The specific reaction between recombinant pseudotyped lentiviral particles and serum detected by ELISA; ^***^p < 0.001. **E**. Fluorescence images of HEK293T cells at 72h post-transfected with recombinant lentivirus particles. A:Control; b:VSV-G/LV-eGFP; c:VSV-G/LV-RABV-G; d: RABV-G/LV-eGFP; e: RABV-G/LV-RABV-G. **F.** The expression of RABV-G mediated by recombinant pseudotyped lentivirus in HEK293T cells.

Firstly, the recombinant plasmids pCMV-RABV-G and pLV-RABV-G were constructed. The restriction enzyme digestion analysis for them with *Nhe* I and *Mlu* I were shown in Fig 2A and 2B. Subsequently, recombinant plasmids were transfected and the rabies virus RABV-G were expression in HEK293T cells transfected with pCMV-RABV-G and pLV-RABV-G, which suggested that recombinant expression vectors were constructed and expressed successfully(S2A Fig). Then, four recombinant lentiviral particles depicted in Fig 1 and the rabies virus RABV-G were expression in HEK293T cells transfected with pCMV-RABV-G and pLV-RABV-G. The VSV-G/LV-eGFP and VSV-G/LV-RABV-G utilize VSV-G as the envelope protein. The viral genome of RABV-G/LV-eGFP contains an eGFP reporter gene expression cassette. The VSV-G/LV-RABV-G includes the target gene RABV-G and an eGFP reporter gene expression cassette linked via an IRES sequence. The RABV-G/LV-eGFP and RABV-G/LV-RABV-G employ RABV-G as the envelope protein. The viral genome of RABV-G/LV-eGFP contains an eGFP reporter gene expression cassette. The RABV-G/LV-RABV-G contains both the target gene RABV-G and an eGFP reporter gene expression cassette linked via an IRES sequence. After transfected 24hrs, the GFP expression in HEK293T cells were observed by fluorescence microscope (S2B Fig), which suggested that plasmid co-transfection was successful.

To determine whether the prepared RABV-G/LV-eGFP and RABV-G/ LV-RABV-G recombinant lentiviral vectors correctly carry the RABV-G antigen, we detected their antigen specificity using western blot and ELISA. The western blot results showed that specific RABV-G bands appeared at approximately 65 kDa in the culture supernatants after transfection (Fig 2C), which indicated that the recombinant lentiviral vectors VSV-G/LV-RABV-G and RABV-G/LV-RABV-G could mediate the expression of the target protein RABV-G in HEK293T cells. In contrast, the control VSV-G/LV-eGFP virus and normal cell culture supernatants did not show positive reaction bands. The ELISA results with diluted the purified corresponding recombinant lentivirus at a ratio of 1:100 as coating antigen showed that when detecting rabies virus antibody-positive serum with RABV-G/LV-eGFP pseudotyped recombinant lentivirus, the mean OD value was 0.691 ± 0.001, while the mean OD value for detecting rabies virus antibody-negative serum was 0.065 ± 0.001. When detecting the two types of serum with RABV-G/LV-RABV-G recombinant lentivirus, the mean OD values were 0.822 ± 0.020 and 0.064 ± 0.001, respectively. When detecting the two types of serum with VSV-G/LV-eGFP recombinant lentivirus, the mean OD values were 0.064 ± 0.001 and 0.065 ± 0.001, respectively(Fig 2D). These results indicate that the RABV-G/LV-eGFP and RABV-G/LV-RABV-G pseudotyped recombinant lentivirus correctly carry the RABV-G antigen and can both produce specific immune reactions with rabies virus antibody-positive serum.

The recombinant lentiviral particles, VSV-G/LV-eGFP, VSV-G/LV-RABV-G, RABV-G/LV-eGFP, and RABV-G/LV-RABV-G were respectively transduced into HEK293T cells. After 72 hrs of culture, observations were made under a fluorescence microscope. The fluorescence protein expression results showed that the prepared recombinant lentiviruses could successfully infect HEK293T cells (Fig 2E). The Western blot results (Fig 2F) indicated that recombinant lentivirus, VSV-G/LV-RABV-G and RABV-G/LV-RABV-G, could mediate the expression of the target protein RABV-G in HEK293T cells.

Additionally, we determined infection titers of recombinant lentivirus to analyze their efficiency. The mean number of fluorescent cells were recorded at 72hrs post-transduced with cultural supernatant diluted at a 10-fold ratio from recombinant lentivirus. In HEK293T cells, the titer of VSV-G/LV-eGFP recombinant lentivirus infection was 1.3 × 10^7^ TU/mL, and that of VSV-G/LV-RABV-G recombinant lentivirus infection was 1.1 × 10^7^ TU/mL. The titer of RABV-G/LV-eGFP recombinant lentivirus infection was 2.1 × 10^6^ TU/mL, and that of RABV-G/LV-RABV-G recombinant lentivirus infection was 1.7 × 10^6^ TU/mL(S3 Fig).

### 3.2 Neutralization test based on RABV pseudotyped recombinant lentivirus

**Fig 3:**
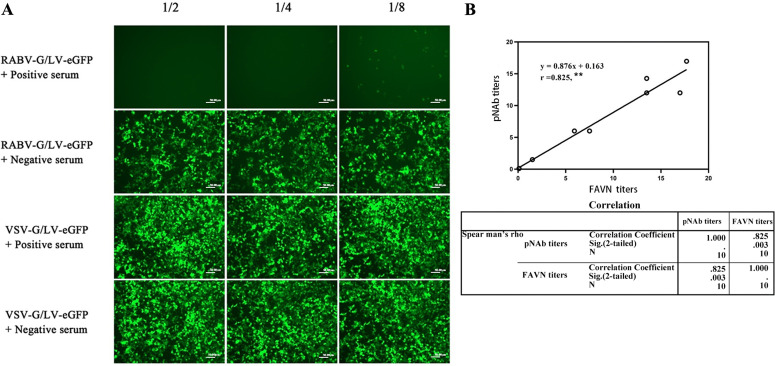
Neutralization assay with the RABV G-pseudotyped recombinant lentivirus. The immunized mouse sera were two fold serially diluted with PBS and incubated with the indicated pseudotyped recombinant lentivirus. The mixture was added to 293T cells. After incubation for 48hs, the serum neutralizing activity against RABV was assessed by the decrease in GFP expression. **A**.Neutralization assay using RABV-G/LV-eGFP and VSV-G/LV-eGFP recombinant lentivirus. **B**. Correlation analysis of recombinant pseudotyped lentivirus neutralization test and FAVN for serum neutralizing antibody titers from vaccinated mice, A high r_s_ value (r_s_ = 0.825, ^**^p < 0.01) indicated a strong positive linear correlation.

To identify whether the prepared RABV-G/LV-eGFP recombinant lentivirus can serve as a pseudovirus for detecting rabies virus-neutralizing antibody titers, we used a known positive serum with a rabies virus-neutralizing antibody titer of 1.5 IU/mL to verify. The serum was serially diluted and incubated with the RABV-G/LV-eGFP recombinant lentivirus, followed by infection of HEK293T cells. The neutralization effect was judged based on subsequent fluorescence protein expression. The results (Fig 3A) showed that positive serum diluted 1:2 and 1:4 completely inhibited the infection ability of the RABV-G/LV-eGFP recombinant lentivirus. The serum diluted 1:8 inhibited most of the infection ability, while negative serum had no inhibitory effect. Meanwhile, the infection ability of VSV-G/LV-eGFP recombinant lentivirus was not inhibited by either positive or negative rabies virus-neutralizing antibody serum. This indicates that the RABV-G/LV-eGFP recombinant lentivirus can undergo specific neutralization reactions with rabies virus-neutralizing antibodies and can be used to detect rabies virus-neutralizing antibody titers.

We then used the RABV recombinant lentiviral pseudovirus neutralization test to detect the neutralizing antibody titers in unknown serum samples, and also measured the neutralizing antibody titers using the fluorescent antibody virus neutralization (FAVN) method. Correlation analysis of the neutralizing antibody titer results from the two methods showed a strong positive correlation (*r* = 0.825), indicating that the RABV pseudotyped recombinant lentivirus neutralization test can effectively determine the serum rabies virus-neutralizing antibody titers (Fig 3B).

### 3.3 Purification of recombinant lentivirus particles using sucrose density gradient ultracentrifugation

**Fig 4:**
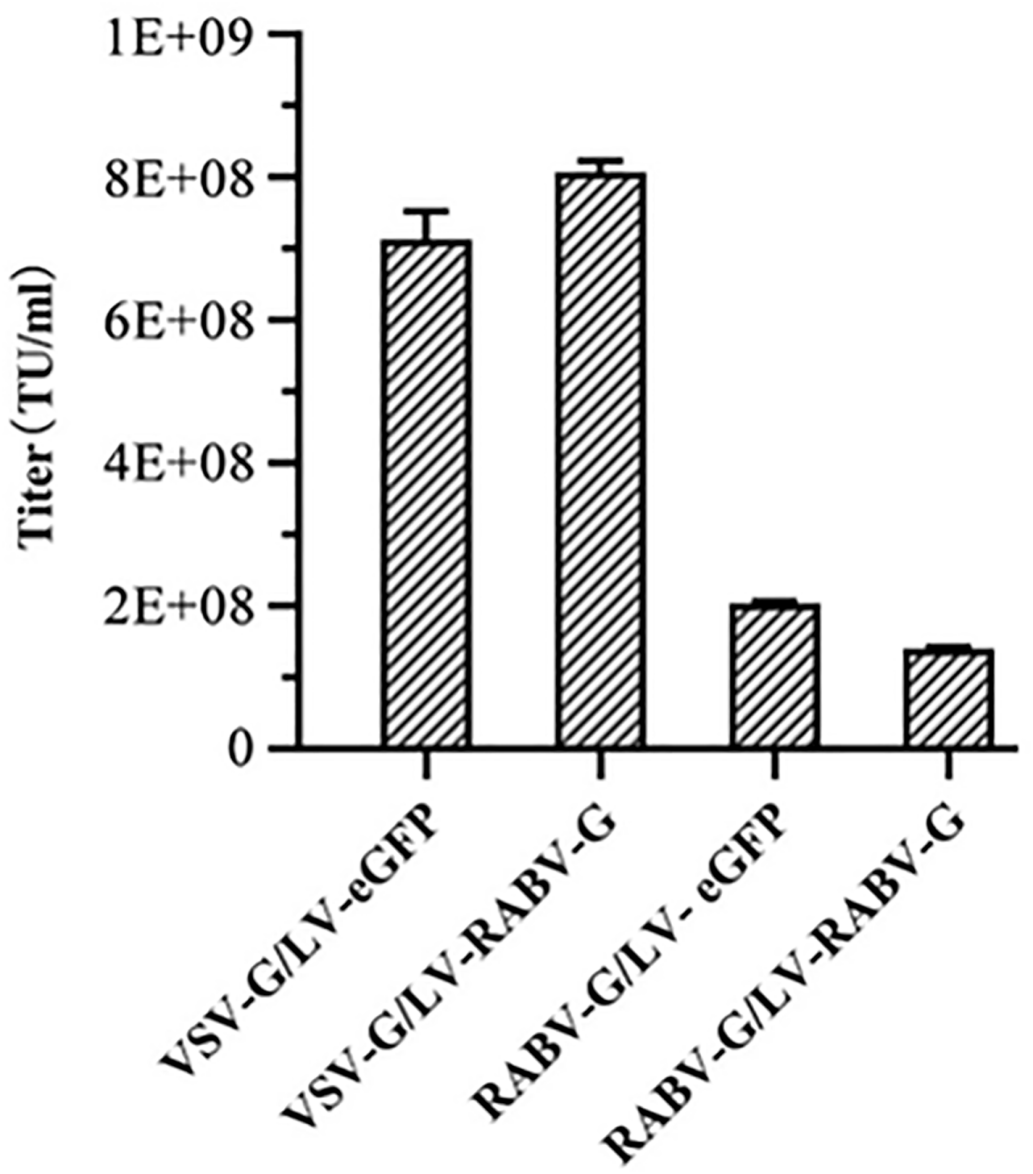
Purification and titers determination of RABV G-pseudotyped recombinant lentivirus. After transfection with plasmids for 48 hours, purify the viral particles packaged by HEK293T cells using sucrose density gradient centrifugation and measure their concentration by qPCR.

Forty-eight hours after co-transfecting HEK293T cells with plasmids, the supernatant was collected and the recombinant lentivirus particles were purified using sucrose density gradient ultracentrifugation. After ultracentrifugation, a bright target band was observed between the 40% and 60% sucrose solutions. The target band was recovered to obtain the purified recombinant lentivirus. The titer of the purified recombinant lentivirus was determined by qPCR (Fig 4). After purificated, the titers of recombinant lentivirus RABV-G/LV-eGFP and RABV-G/LV-RABV-G separately were 1.90 × 10^8^TU/mL and 1.58 × 10^8^TU/mL, while the titers of the other two, VSV-G/LV-eGFP and VSV-G/LV-RABV-G, were 7.1 × 10^8^TU/mL and 8.2 × 10^8^TU/ mL. All of which were higher than un-purified recombinant lentivirus (S3 Fig)

### 3.4 Rapid and sustained induction of both Anti-RABV-G and neutralizing antibodies in mice by recombinant lentivirus VSV-G/LV-RABV-G and RABV-G/LV-RABV-G

**Fig 5:**
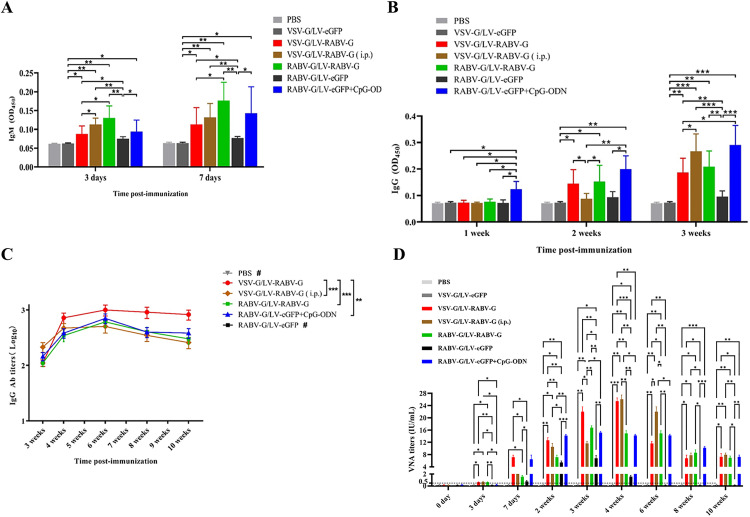
Evaluation of anti-RABV-G immunogenicity of pseudotyped recombinant lentivirus in mice after one-dose immunization. **A**. Detection of immunized serum anti-RABV-G IgM antibody levels in inoculated mice at 3^th^ and 7^th^ day. **B**. Detection of immunized serum anti-RABV-G IgG antibody levels in inoculated mice at 1^st^, 2^nd^ and 3^rd^ week. **C.** Detection of sustained serum anti-RABV-G IgG antibody titers in different groups. **D**. Detection of immunized serum neutralizing antibody titers in different groups. ^***^p < 0.001, ^**^p < 0.01, ^*^p < 0.05, #: The antibody titers did not reach detectable levels.

The immunogenicity of each RABV-G recombinant lentivirus was assessed in mice. Serum anti-RABV-G IgM antibody levels were measured at 3^rd^ day and 7^th^ day after a single immunization. The results showed that the serum IgM antibody levels in the VSV-G/LV-RABV-G, VSV-G/LV-RABV-G(i.p.), and RABV-G/LV-RABV-G groups were significantly higher than those in the control VSV-G/LV-eGFP group at 3 days post-immunization and continued to increase in these groups at 7days post-immunization (Fig 5A). Regarding serum IgG antibody levels post-immunization, no significant anti-RABV-G antibody levels were detected in the VSV-G/LV-RABV-G group and the RABV-G/LV-RABV-G group one week after a single immunization. However, anti-RABV-G antibody levels in the VSV-G/LV-RABV-G group and the RABV-G/LV-RABV-G group increased significantly compared to the control VSV-G/LV-eGFP group two weeks after a single immunization and continued to rise in these two groups three weeks after a single immunization (Fig 5B). Additionally, anti-RABV-G antibody levels in the VSV-G/LV-RABV-G(i.p.) group increased significantly at three weeks, showing a statistical difference compared to the VSV-G/LV-eGFP group and being significantly higher than those in the VSV-G/LV-RABV-G group.

To investigate the dynamics and duration of anti-RABV-G IgG antibody levels in mice, the serum IgG antibody titers were measured at different time points post-immunization. The results showed that anti-RABV-G IgG antibody titers increased, reaching a high point at 4 weeks after a single immunization and peaking at 6weeks in the VSV-G/LV-RABV-G group and the RABV-G/LV-RABV-G group. The serum IgG antibody titers in the VSV-G/LV-RABV-G (i.p.) group peaked at 4 weeks post-immunization (Fig 5C). In these groups, the serum IgG antibody titers remained at high levels at 10 weeks. Notably, the average serum IgG antibody titer in the VSV-G/LV-RABV-G group was significantly higher during the antibody persistence period compared to the other positive groups. No IgG antibody titers were detected in the PBS group and the VSV-G/LV-eGFP group.

The serum neutralizing antibody titers were measured at various time points post-immunization using the RABV recombinant lentiviral pseudovirus neutralization test. The results showed that in the VSV-G/LV-RABV-G group, the VSV-G/LV-RABV-G (i.p.) group, and the RABV-G/LV-RABV-G group, neutralizing antibody levels were detectable as early as 3days post-immunization, reaching the rabies vaccine protective unit of 0.5 IU/mL, and maintained high neutralizing antibody levels at more than 0.5 IU/mL up to 10weeks post-immunization (Fig 5D). In contrast, the PBS group and the VSV-G/LV-eGFP group did not produce detectable neutralizing antibodies at any time point.

### 3.5 Induction of both Anti-RABV-G and neutralizing antibodies by RABV-G/LV-eGFP recombinant lentivirus

The recombinant lentivirus RABV-G/LV-eGFP alone elicited low levels of anti-RABV-G antibodies and neutralizing antibodies in mice after a single immunization. However, when combined with CPG-ODN as an adjuvant, the recombinant lentivirus RABV-G/LV-eGFP showed a significant increase in anti-RABV-G IgM antibody levels at 3days post-immunization, which continued to rise at 7days post-immunization and even to 3weeks (Fig 5A and 5B). Additionally, this combination elicited higher serum IgG antibody and neutralizing antibody titers, exceeding 0.5IU/mL at 7 days post-immunization and maintaining these levels for at least 10weeks post-immunization (Fig 5C and 5D).

### 3.6 Enhanced immunogenicity with two-dose immunization

**Fig 6:**
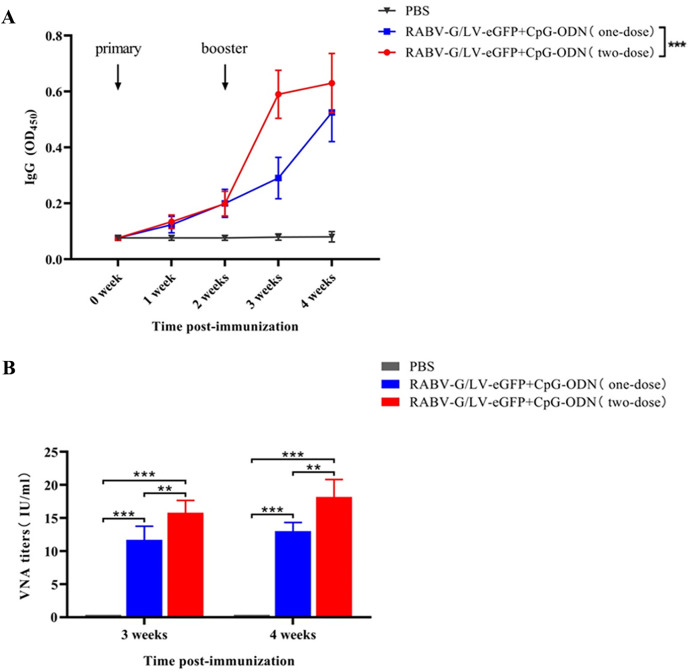
Evaluation of anti-RABV-G immunogenicity of each pseudotyped recombinant lentivirus in mice with different immunization mode. **A.** Detection of serum anti-RABV IgG antibody levels in different groups of mice after one or two-dose immunization. **B**. Detection of neutralizing antibody titers in serum from different groups after one or two-dose immunization at 3^nd^ and 4^th^ week. ^***^p < 0.001, ^**^p < 0.01.

Mice in the RABV-G/LV-eGFP + CPG-ODN group received a booster immunization two weeks after the initial immunization. The serum anti-RABV-G IgG antibody levels were measured, as shown in Fig 6A. One week after the booster immunization, the IgG antibody levels in the RABV-G/LV-eGFP + CPG-ODN (two-dose) group significantly increased. Additionally, the serum neutralizing antibody titers in this group were significantly higher than those in the single-dose group. Two weeks after the booster immunization, the neutralizing antibody titers in the RABV-G/LV-eGFP + CPG-ODN (two-dose) group further increased and remained significantly higher than the neutralizing antibody titers in the single-dose group (Fig 6B).

## 4. Discussion

Rabies continues to be a significant public health threat globally, necessitating ongoing research into improved vaccination strategies. The lentiviral vector vaccines show a good application prospect because of their efficient gene delivery mechanism, biosafety and early and lasting immune stimulation ability in the development of vaccines [23,24]. We have used our lentiviral-packaged system dependent on HEK293T cells for studying rabies virus and COVID-19 subunit vaccine and confirmed to be effective [18,25]. Till now, pseudotyped lentiviral vectors had been used in vaccine development for a variety of infectious diseases, which represents a promising advancement in the field, offering potential enhancements over traditional vaccines in terms of safety, efficacy, and ease of administration [26–28]. And it provides reference and basis for our long-term work in the development of rabies vaccine.

In our study we constructed three recombinant lentivirus vaccines: VSV-G/LV-RABV-G, RABV-G/LV-eGFP and RABV-G/LV-RABV-G based on rabies virus G protein and lentiviral vector system, each of which was engineered with specific attributes aimed at boosting immunogenicity and simplifying vaccine delivery. Firstly, we obtained the target gene of rabies virus G protein and successfully constructed the recombinant lentivirus VSV-G/LV-RABV-G. The recombinant lentivirus was detected to mediate the expression of G protein in HEK293T cells, which laid the foundation for the development of rabies recombinant lentivirus vector vaccine. Early immune response and protection are very important for the body to resist the attack of the rabies virus [29]. Rabies viruses belong to the rhabdoviridae family like vesicular stomatitis viruses, and have similar infective properties and membrane fusion ability due to the characteristics of their surface glycoproteins. In order to increase the immunogenicity of the recombinant lentiviral vector, we used the recombinant RABV-G plasmid replace VSV-G plasmid and obtained the recombinant lentiviral particle RABV-G/LV-eGFP and RABV-G/LV-RABV-G. It is worth mentioning that RABV-G/LV-RABV-G possesses two types of G protein antigens, in which one G protein antigen was directly carried in the recombinant viral particle as the envelope protein and another G protein antigen is expressed inside the cell by gene delivery. Therefore, the RABV-G/LV-RABV-G lentiviral particle simultaneously uses two antigens to stimulate the body. Theoretically, after vaccine immunization, the G protein antigen directly carried in the recombinant virus particle is first recognized by immune cells and activated the immune system, and then the G protein antigen continuously expressed in the cell through gene transfer activates the immune system again to produce specific immune response, which would improve the immunity of the vaccine.

Usually after vaccination, the IgM antibodies plays a key role before IgG antibodies products in the body. In the early stage of B-cell-mediated humoral immunity, when IgG antibodies are insufficient, IgM antibodies are mainly used to clear pathogens [30,31]. In terms of the protective power of vaccines, the recombinant lentivirus vaccines VSV-G/LV-RABV-G and RABV-G/LV-RABV-G showed sufficient protective ability from IgM 3 days after a single immunization. IgM antibodies are generally considered to have a limited role in clearing highly neurotropic viruses, like rabies [32]. However, recent studies have shown that IgM antibodies induced by immune stimulation play an important role in limiting the transmission of some neurotropic viruses due to its early generation time point, and its high number of antigen-binding sites enables IgM antibodies to neutralize the virus more effectively [33]. Michael *et al.* found that IgM produced in the early stage of immunity could limit the spread of West Nile virus in the blood and central nervous system of mice, and mice with high IgM antibody levels had lower mortality in early viral infection [31]. Studies have shown that IgG antibodies induced by traditional inactivated rabies virus vaccine can produce effective protection, while naturally occurring IgM antibodies or IgM antibodies induced by rabies virus inactivated vaccine cannot protect mice in challenge [34]. In this study, the RABV-G/LV-eGFP vaccine produce an immune response stimulated by the G protein antigen directly, and its immune principle was similar to that of the inactivated rabies virus vaccine. RABV-G/LV-eGFP vaccine assisted by CpG-ODN adjuvant produced a higher level of IgM antibody at 3days, but no neutralizing antibody production was detected, which indicated that the RABV-G/LV-eGFP vaccine was the same as the inactivated rabies virus vaccine and the IgM antibody induced by it could not produce a protective effect on the body at 3days. However, another study from Dorfmeier *et al.* confirmed that IgM induced by uninactivated rabies virus attenuated strain can play a direct protective role in the attack protection against rabies virus. After immunizing normal mice with rabies virus attenuated strain, IgM antibodies were produced at 3 days and had a protective effect in the attack [35]. The combination of the neutralizing antibody levels of the VSV-G/LV-RABV-G and the RABV-G/LV-RABV-G vaccine further indicated that the recombinant lentiviral vector vaccine expressing G protein antigen through gene delivery can induce IgM antibodies after 3days and protect the body.

We also studied the effect of different injection sites on the immune efficacy of recombinant lentiviral vector vaccines. The results showed that neutralizing antibody production was induced in mice 3days after a single intramuscular injection of VSV-G/LV-RABV-G and RABV-G/LV-RABV-G vaccine immunization, and the high antibody titers sustained for 10 weeks. Moreover, mice injected intraperitoneally with the VSV-G/LV-RABV-G vaccine showed similar levels of neutralizing antibodies as the intramuscular group. These results suggest that both VSV-G/LV-RABV-G and RABV-G/LV-RABV-G vaccines can provide early and good immune protection with a single injection, and can be used directly for single-dose and emergency post-exposure immunization without adjuvant assistance.

The proper use of adjuvants can effectively impart superior immunity of vaccine [36,37]. CPG-ODN 1018 can significantly enhance the humoral immune response of mice to a variety of antigens such as influenza hemagglutinin and HIV glycoprotein, and the significant increase of IgG2a in mice is mainly due to the promoting effect of CpG sequence on Th-1 immune response [38,39]. The main receptor of CpG-ODN is TLR-9, and the level of TLR-9 expressed in mice and humans is quite different, and the cells that mainly express TLR-9 are also different. The activation of TLR-9 in mice usually leads to the secretion of more antibodies and cytokines [38]. CpG-ODN 1018 simultaneously contains two different sequences that are active against TLR-9 in mice and primates including humans [40]. In combination with HBV vaccine, CpG-ODN 1018 significantly increases the level of HBV-specific antibodies in primates. The antibody titer was 45 times higher than that of hepatitis B vaccine assisted with alum adjuvant, demonstrating its powerful adjuvant activity in primates. At the same time, CpG-ODN 1018 and hepatitis B virus vaccine have been used in clinical trials, which can induce earlier protective immune response in healthy people compared with hepatitis B vaccine using aluminum adjuvant [41], and can also achieve good immune effect in the elderly and people with immune system injury [42]. In our study, CpG-ODN 1018 adjuvant was used to assist RABV-G/LV-eGFP vaccine to induce early and lasting humoral immune response and neutralizing antibody level in mice after a single injection, and stronger protective ability could be obtained after enhanced immunity. Additionally, enhanced immunization of two dose with RABV-G/LV-eGFP and CpG-ODN 1018 adjuvant showed that the IgG antibody levels and neutralizing antibody levels were significantly higher than those of the single immunization group. This study provided an experimental basis for the formulation of RABV-G/LV-eGFP vaccine immunization strategy, and proved that CpG-ODN 1018 adjuvant can be effectively applied to RABV-G/LV-eGFP recombinant lentiviral vector vaccination.

Currently, the fluorescent Antibody Virus neutralization test (FAVN) and rapid fluorescence focus inhibition test (RFFIT) recommended by the World Health Organization (WHO) and the Office for International Veterinary Diseases (OIE) are commonly used for the detection of rabies virus neutralizing antibody titers. Both methods need to operate rabies virus, fix the infected cells and stain with anti-rabies nuclear protein fluorescent antibody, which is not safety and usually carried out in BSL-3 laboratory [43]. Only a few laboratories in China can do the neutralizing antibodies test. In order to avoid exposure to live viruses, people have tried to use recombinant viruses with fluorescent proteins instead of direct viral fluorescent staining [44,22].We established the detection method of rabies virus neutralizing antibody based on RABV-G/LV-eGFP recombinant Lentivirus (RABV pseudovirus). The results of the RABV neutralizing antibody titer were positively correlated with those by FAVN test, which indicated RABV pseudovirus neutralization test can effectively determine the rabies virus neutralizing antibody titer in serum. The important thing is that the RABV pseudovirus used can be prepared and used in the BSL-2 laboratory, with high safety. The degree of virus neutralization reaction can be quickly and directly observed through the GFP reporter protein. It can replace the live virus detection method to detect the rabies virus neutralization antibody titer because of economy and convenience, and has good application value.

## 5. Conclusion

Currently, lentiviral vector vaccines are still in clinical trials or preclinical studies and have not yet been officially approved for market release. Major progress has been made in areas such as chronic HBV, tumor immunotherapy, and viral infection prevention. Their technological advantages, such as long-lasting immunity and strong cellular response, along with the optimization of production processes, like the use of stable cell lines and cost-effective production, lay the foundation for future vaccine launches. A non-integrative lentiviral vector vaccine (LV-LHBs) developed by teams including Fudan University successfully induced strong HBV-specific T-cell responses in mouse models, significantly lowering serum HBsAg levels and viral load. Safety and preliminary efficacy were also observed in two inactive HBsAg carriers [45]. Our study underscores that recombinant pseudotyped lentiviral vector vaccines encoding the RABV-G antigen hold promise as effective alternatives to traditional rabies vaccines. They induce rapid and durable immune responses in animal models, offering potential advantages in terms of efficacy, safety, and practicality. Continued research and development efforts are warranted to further refine these vaccines and evaluate their efficacy in human populations, aiming to mitigate the global burden of rabies infections.

## Supporting information

S1 Fig Schematic diagram of recombinant expression vector construction. A. Recombinant expression vector pLV-RABV-G construction. (B).Recombinant expression vector pCMV-RABV-G construction.
(TIF)

S2 Fig Recombinant expression vector construction and virus packaging effect verification. (**A**) Analysis of Rabies virus RABV-G expression in HEK293T cells transfected with recombinant vectors pCMV-RABV-G and pLV-RABV-G, which suggested that the RABV-G recombinant expression vectors were constructed successfully. (**B**) Fluorescence images of HEK293T cells at 24hrs post-transfected with the different RABV-G recombinant exprssion vectors and related package plasmids, which suggested that the recombinant expression vectors were constructed and transfected successfully. A: Control; B: psPAX2, pLV-eGFP and pCMV-VSV-G; C: psPAX2, pLV-RABV-G and pCMV-VSV-G; D: psPAX2, pLV-eGFP and pCMV-RABV-G; E: psPAX2, pLV-RABV-G and pCMV-RABV-G.
(TIF)

S3 Fig Detection of transduction unites of recombinant lentivirus. Fluorescence images of HEK293T cells at 72hrs post-transducted with cultural supernatant diluted at a 10-fold ratio from recombinant lentivirus. In HEK293T cells, the titer of VSV-G/LV-eGFP recombinant lentivirus infection was 1.3 × 10^7^ TU/ml, and that of VSV-G/LV-RABV-G recombinant lentivirus infection was 1.1 × 10^7^ TU/ml. The titer of RABV-G/LV-eGFP recombinant lentivirus infection was 2.1 × 10^6^ TU/ml, and that of RABV-G/LV-RABV-G recombinant lentivirus infection was 1.7 × 10^6^ TU/ml.
(TIF)

S1 Data Raw data for Fig 2D. the raw data of the ELISA results with diluted the purified correspongding recombinant lentivirus at a ratio of 1:100 as coating antigen showed that when detecting rabies virus antibody-positive serum. Raw data for Fig 4. the raw data of purification and titers determination of RABV G-pseudotyped recombinant lentivirus by qPCR. Raw data for Fig 5. the raw data of the ELISA results for anti-RABV-G immunogenicity of pseudotyped recombinant lentivirus in mice after one-dose immunization. Raw data for Fig 6. the raw data of the ELISA results for anti-RABV-G immunogenicity of each pseudotyped recombinant lentivirus in mice with different immunization mode.
(RAR)

## References

[1] Kumar A, Bhatt S, Kumar A, Rana T. Canine rabies: an epidemiological significance, pathogenesis, diagnosis, prevention, and public health issues. Comp Immunol Microbiol Infect Dis. 2023;97:101992. doi: 10.1016/j.cimid.2023.101992 37229956

[2] Jin J. Rabies. JAMA. 2023;329(4):350. doi: 10.1001/jama.2022.22254 36692559

[3] Knobel DL, Cleaveland S, Coleman PG, Fèvre EM, Meltzer MI, Miranda MEG, et al. Re-evaluating the burden of rabies in Africa and Asia. Bull World Health Organ. 2005;83(5):360–8. 15976877 PMC2626230

[4] Fisher CR, Schnell MJ. New developments in rabies vaccination. Rev Sci Tech. 2018;37(2):657–72. doi: 10.20506/rst.37.2.2831 30747119

[5] Li J, Liu Q, Liu J, Wu X, Lei Y, Li S, et al. An mRNA-based rabies vaccine induces strong protective immune responses in mice and dogs. Virol J. 2022;19(1):184. doi: 10.1186/s12985-022-01919-7 36371169 PMC9652961

[6] Ng WM, Fedosyuk S, English S, Augusto G, Berg A, Thorley L, et al. Structure of trimeric pre-fusion rabies virus glycoprotein in complex with two protective antibodies. Cell Host Microbe. 2022;30(9):1219–1230.e7. doi: 10.1016/j.chom.2022.07.014 35985336 PMC9605875

[7] Astray RM, Jorge SAC, Pereira CA. Rabies vaccine development by expression of recombinant viral glycoprotein. Arch Virol. 2016;162(2):323–32. doi: 10.1007/s00705-016-3128-9 27796547

[8] Lundstrom K. RNA viruses as tools in gene therapy and vaccine development. Genes (Basel). 2019;10(3):189. doi: 10.3390/genes10030189 30832256 PMC6471356

[9] Frietze KM, Peabody DS, Chackerian B. Engineering virus-like particles as vaccine platforms. Curr Opin Virol. 2016;18:44–9. doi: 10.1016/j.coviro.2016.03.001 27039982 PMC4983494

[10] Ku M-W, Charneau P, Majlessi L. Use of lentiviral vectors in vaccination. Expert Rev Vaccines. 2021;20(12):1571–86. doi: 10.1080/14760584.2021.1988854 34620025

[11] Bona R, Michelini Z, Mazzei C, Gallinaro A, Canitano A, Borghi M, et al. Safety and efficiency modifications of SIV-based integrase-defective lentiviral vectors for immunization. Mol Ther Methods Clin Dev. 2021;23:263–75. doi: 10.1016/j.omtm.2021.09.011 34729374 PMC8526422

[12] Lemiale F, Korokhov N. Lentiviral vectors for HIV disease prevention and treatment. Vaccine. 2009;27(25–26):3443–9. doi: 10.1016/j.vaccine.2009.01.055 19201386

[13] Norton TD, Miller EA. Recent advances in lentiviral vaccines for HIV-1 infection. Front Immunol. 2016;7:243. doi: 10.3389/fimmu.2016.00243 27446074 PMC4914507

[14] Gallinaro A, Borghi M, Bona R, Grasso F, Calzoletti L, Palladino L, et al. Integrase defective lentiviral vector as a vaccine platform for delivering influenza antigens. Front Immunol. 2018;9. doi: 10.3389/fimmu.2018.00171 PMC5807328 29459873

[15] Du Y, Zhang S, Zhang Z, Miah KM, Wei P, Zhang L, et al. Intranasal lentiviral vector-mediated antibody delivery confers reduction of SARS-CoV-2 infection in elderly and immunocompromised mice. Front Immunol. 2022;13. doi: 10.3389/fimmu.2022.819058 PMC9072863 35529866

[16] Iglesias MC, Frenkiel M-P, Mollier K, Souque P, Despres P, Charneau P. A single immunization with a minute dose of a lentiviral vector-based vaccine is highly effective at eliciting protective humoral immunity against West Nile virus. J Gene Med. 2006;8(3):265–74. doi: 10.1002/jgm.837 16308885

[17] Mao Y, Yan R, Li A, Zhang Y, Li J, Du H, et al. Lentiviral vectors mediate long-term and high efficiency transgene expression in HEK 293T cells. Int J Med Sci. 2015;12(5):407–15. doi: 10.7150/ijms.11270 26005375 PMC4441065

[18] Li Q, Yan R, Bai N, Tan Z, Yu Q, Su H, et al. Immunogenicity and antigenicity of the ectodomain of rabies virus glycoprotein stably expressed in HEK293T cells. Int J Med Sci. 2023;20(10):1282–92. doi: 10.7150/ijms.87134 37786447 PMC10542018

[19] Kurz J, Vogel I, Gerstl F, Dostal V. Comparative studies of two potency tests for antirabies serum: neutralization test in mice (MNT) and rapid fluorescent focus inhibition test (RFFIT). Dev Biol Stand. 1986;64:99–107. 3539671

[20] Mo H, Chen Q, Zhang Z, Lin G, Wang Y, Mo L, et al. Development of a blocking ELISA for evaluating neutralizing antibodies in human and canine serum based on rabies virus glycoprotein epitope I. Int J Biol Macromol. 2025;301:140275. doi: 10.1016/j.ijbiomac.2025.140275 39863206

[21] Wu G, McElhinney LM, Goharriz H, Amaya-Cuesta J, Fooks AR, Banyard AC. A simplified method for measuring neutralising antibodies against rabies virus. J Virol Methods. 2023;319:114769. doi: 10.1016/j.jviromet.2023.114769 37391076

[22] Yang DK, Kim HH, Park YR, Yoo JY, Park Y, Park J, et al. Generation of a recombinant rabies virus expressing green fluorescent protein for a virus neutralization antibody assay. J Vet Sci. 2021;22(4):e56. doi: 10.4142/jvs.2021.22.e56 34313041 PMC8318786

[23] Nemirov K, Bourgine M, Anna F, Wei Y, Charneau P, Majlessi L. Lentiviral vectors as a vaccine platform against infectious diseases. Pharmaceutics. 2023;15(3):846. doi: 10.3390/pharmaceutics15030846 36986707 PMC10053212

[24] Ku M-W, Charneau P, Majlessi L. Use of lentiviral vectors in vaccination. Expert Rev Vaccines. 2021;20(12):1571–86. doi: 10.1080/14760584.2021.1988854 34620025

[25] Yan R, Liu J, Chen Z, Wan P, Liang T, Li K, et al. Rapid production of COVID-19 subunit vaccine candidates and their immunogenicity evaluation in pigs. Inter J Biol Macromol. 2024;272:132798. doi: 10.1016/j.ijbiomac.2024.132798 38838896

[26] Ferrara F, Del Rosario JMM, da Costa KAS, Kinsley R, Scott S, Fereidouni S, et al. Development of lentiviral vectors pseudotyped with influenza B hemagglutinins: application in vaccine immunogenicity, mAb potency, and sero-surveillance studies. Front Immunol. 2021;12:661379. doi: 10.3389/fimmu.2021.661379 34108964 PMC8182064

[27] Li Q, Huang W, Wang Y. Pseudotyped viruses for mammarenavirus. Adv Exp Med Biol. 2023;1407:279–97. doi: 10.1007/978-981-99-0113-5_15 36920703

[28] Steffen I, Simmons G. Pseudotyping viral vectors with emerging virus envelope proteins. Curr Gene Ther. 2016;16(1):47–55. doi: 10.2174/1566523216666160119093948 26785737

[29] Guo Y, Duan M, Wang X, Gao J, Guan Z, Zhang M. Early events in rabies virus infection-attachment, entry, and intracellular trafficking. Virus Res. 2019;263:217–25. doi: 10.1016/j.virusres.2019.02.006 30772332

[30] Atre T, Phillips RL, Modjarrad K, Regules JA, Bergmann-Leitner ES. Development and characterization of a Zaire Ebola (ZEBOV) specific IgM ELISA. J Immunol Methods. 2019;468:29–34. doi: 10.1016/j.jim.2019.03.008 30910536

[31] Diamond MS, Sitati EM, Friend LD, Higgs S, Shrestha B, Engle M. A critical role for induced IgM in the protection against West Nile virus infection. J Exp Med. 2003;198(12):1853–62. doi: 10.1084/jem.20031223 14662909 PMC2194144

[32] Begeman L, GeurtsvanKessel C, Finke S, Freuling CM, Koopmans M, Müller T, et al. Comparative pathogenesis of rabies in bats and carnivores, and implications for spillover to humans. Lancet Infect Dis. 2018;18(4):e147–59. doi: 10.1016/S1473-3099(17)30574-1 29100899

[33] Nakayasu M, Hirano M, Muto M, Kobayashi S, Kariwa H, Yoshii K. Development of a serodiagnostic IgM-ELISA for tick-borne encephalitis virus using subviral particles with strep-tag. Ticks Tick Borne Dis. 2018;9(6):1391–4. doi: 10.1016/j.ttbdis.2018.06.010 29960872

[34] Turner GS. Immunoglobulin (IgG) and (IgM) antibody responses to rabies vaccine. J Gen Virol. 1978;40(3):595–604. doi: 10.1099/0022-1317-40-3-595 690611

[35] Dorfmeier CL, Shen S, Tzvetkov EP, McGettigan JP. Reinvestigating the role of IgM in rabies virus postexposure vaccination. J Virol. 2013;87(16):9217–22. doi: 10.1128/jvi.00995-13 23760250 PMC3754079

[36] Zhao T, Cai Y, Jiang Y, He X, Wei Y, Yu Y, et al. Vaccine adjuvants: mechanisms and platforms. Signal Transduct Target Ther. 2023;8(1):283. doi: 10.1038/s41392-023-01557-7 37468460 PMC10356842

[37] Verma SK, Mahajan P, Singh NK, Gupta A, Aggarwal R, Rappuoli R, et al. New-age vaccine adjuvants, their development, and future perspective. Front Immunol. 2023;14:1043109. doi: 10.3389/fimmu.2023.1043109 36911719 PMC9998920

[38] Gupta K, Cooper C. A review of the role of CpG Oligodeoxynucleotides as toll-like receptor 9 agonists in prophylactic and therapeutic vaccine development in infectious diseases. Drugs in R & D. 2008;9(3):137–45. doi: 10.2165/00126839-200809030-00001 18457466

[39] Horner AA, Datta SK, Takabayashi K, Belyakov IM, Hayashi T, Cinman N, et al. Immunostimulatory DNA-based vaccines elicit multifaceted immune responses against HIV at systemic and mucosal sites. J Immunol. 2001;167(3):1584–91. doi: 10.4049/jimmunol.167.3.1584 11466380

[40] Campbell JD, Kell SA, Kozy HM, Lum JA, Sweetwood R, Chu M, et al. A limited CpG-containing oligodeoxynucleotide therapy regimen induces sustained suppression of allergic airway inflammation in mice. Thorax. 2014;69(6):565–73. doi: 10.1136/thoraxjnl-2013-204605 24464743 PMC4558941

[41] Halperin SA, Dobson S, McNeil S, Langley JM, Smith B, McCall-Sani R, et al. Comparison of the safety and immunogenicity of hepatitis B virus surface antigen co-administered with an immunostimulatory phosphorothioate oligonucleotide and a licensed hepatitis B vaccine in healthy young adults. Vaccine. 2006;24(1):20–6. doi: 10.1016/j.vaccine.2005.08.095 16198027

[42] Janssen JM, Heyward WL, Martin JT, Janssen RS. Immunogenicity and safety of an investigational hepatitis B vaccine with a Toll-like receptor 9 agonist adjuvant (HBsAg-1018) compared with a licensed hepatitis B vaccine in patients with chronic kidney disease and type 2 diabetes mellitus. Vaccine. 2015;33(7):833–7. doi: 10.1016/j.vaccine.2014.12.060 25576215

[43] Yu P, Lv X, Shen X, Tang Q, Liang G. Establishment and preliminary application of a rapid fluorescent focus inhibition test (RFFIT) for rabies virus. Virol Sin. 2013;28(4):223–7. doi: 10.1007/s12250-013-3321-x 23913179 PMC8208402

[44] Tang H-B, Lu Z-L, Wei X-K, Zhong Y-Z, Zhong T-Z, Pan Y, et al. A recombinant rabies virus expressing a phosphoprotein-eGFP fusion is rescued and applied to the rapid virus neutralization antibody assay. J Virol Methods. 2015;219:75–83. doi: 10.1016/j.jviromet.2015.03.022 25845623

[45] Zhang Y, Bourgine M, Wan Y, Song J, Li Z, Yu Y, et al. Therapeutic vaccination with lentiviral vector in HBV-persistent mice and two inactive HBsAg carriers. J Hepatol. 2024;80(1):31–40. doi: 10.1016/j.jhep.2023.09.019 37827470

