# Supplementary figures and images for: Novel rabies vaccine candidates development based on pseudotyped lentiviral vectors with rabies virus glycoprotein
Source: PLoS Negl Trop Dis. 2025 Aug 26;19(8):e0013404. doi: 10.1371/journal.pntd.0013404 (PMC12380317; doi:10.1371/journal.pntd.0013404)

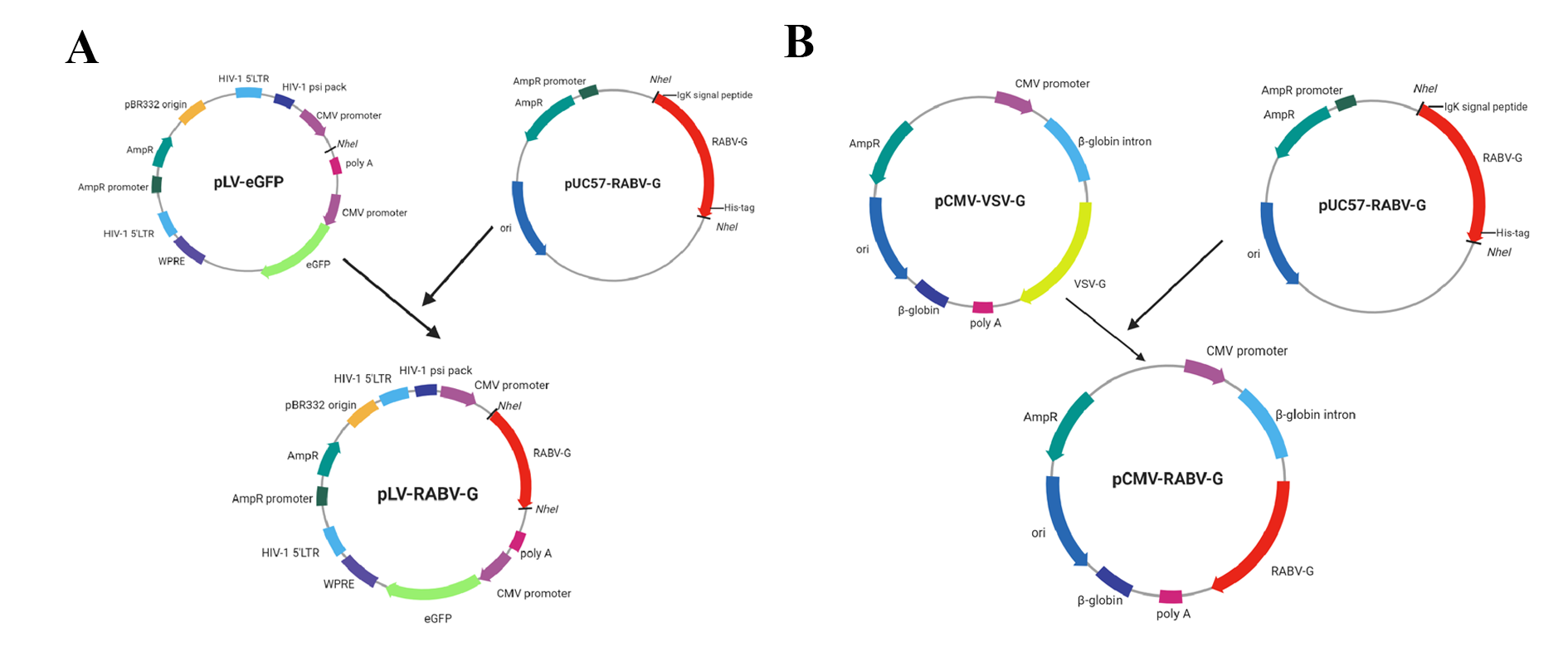

Supplement: S1 Fig — A. Recombinant expression vector pLV-RABV-G construction. (B).Recombinant expression vector pCMV-RABV-G construction. (TIF) [file pntd.0013404.s001.tif]

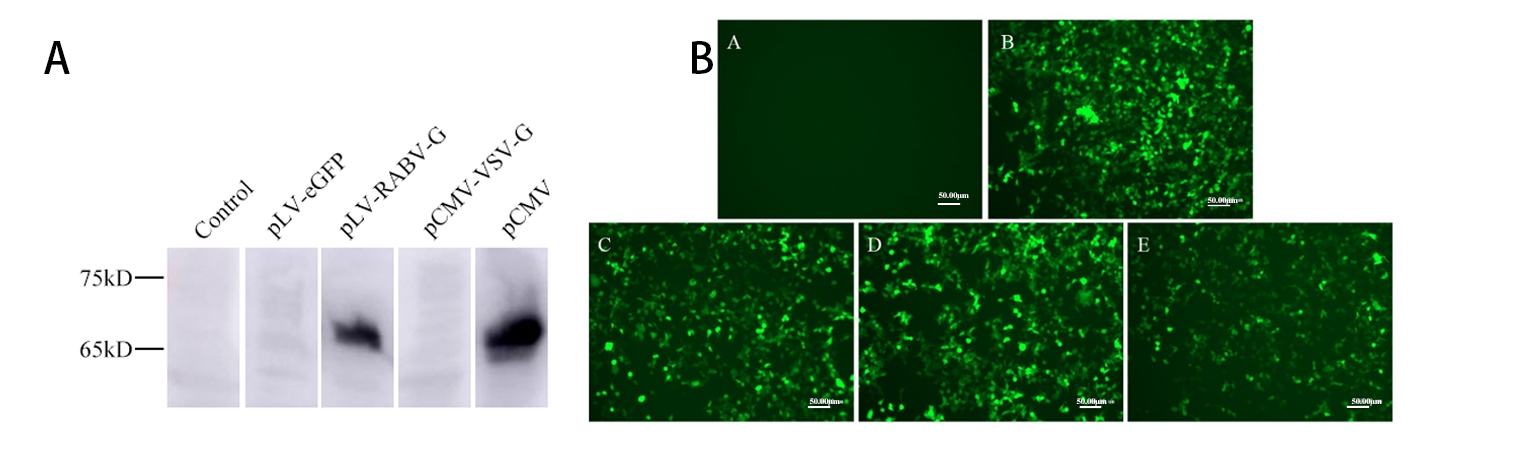

Supplement: S2 Fig — (A) Analysis of Rabies virus RABV-G expression in HEK293T cells transfected with recombinant vectors pCMV-RABV-G and pLV-RABV-G, which suggested that the RABV-G recombinant expression vectors were constructed successfully. (B) Fluorescence images of HEK293T cells at 24hrs post-transfected with the different RABV-G recombinant exprssion vectors and related package plasmids, which suggested that the recombinant expression vectors were constructed and transfected successfully. A: Control; B: psPAX2, pLV-eGFP and pCMV-VSV-G; C: psPAX2, pLV-RABV-G and pCMV-VSV-G; D: psPAX2, pLV-eGFP and pCMV-RABV-G; E: psPAX2, pLV-RABV-G and pCMV-RABV-G. (TIF) [file pntd.0013404.s002.tif]

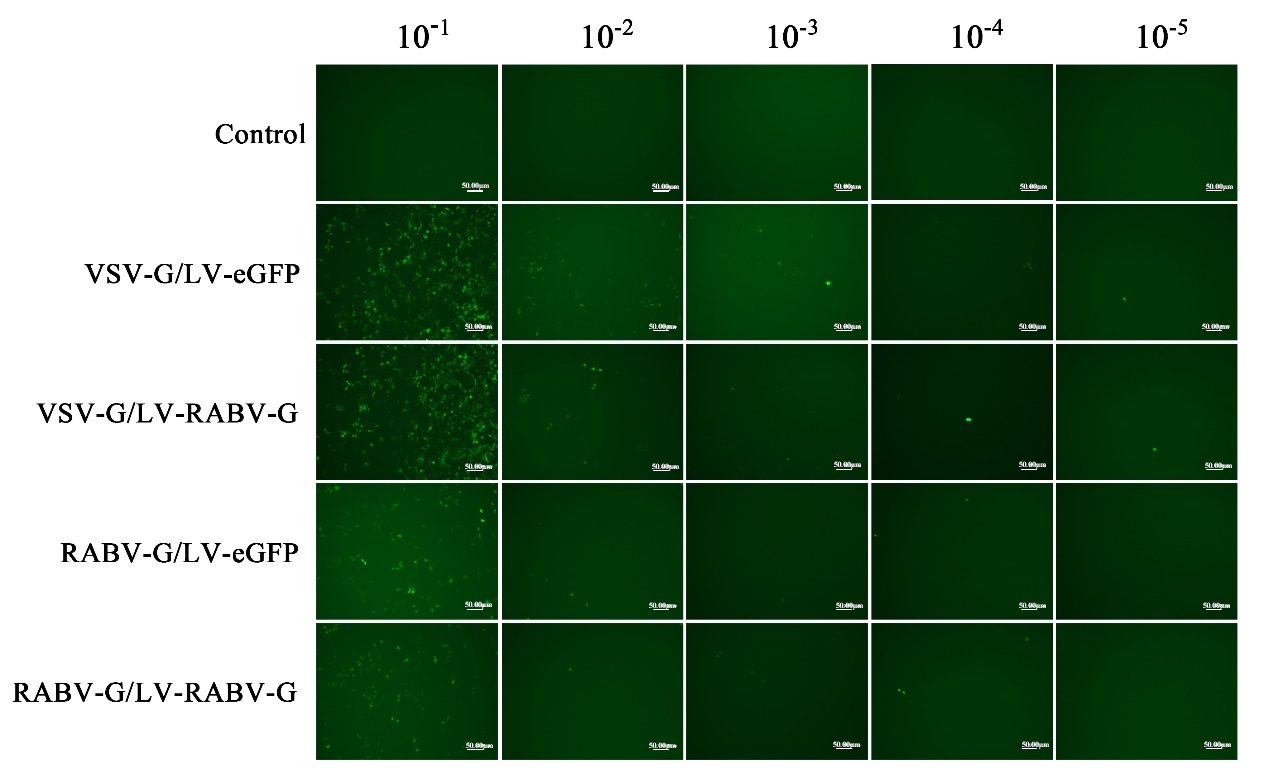

Supplement: S3 Fig — Fluorescence images of HEK293T cells at 72hrs post-transducted with cultural supernatant diluted at a 10-fold ratio from recombinant lentivirus. In HEK293T cells, the titer of VSV-G/LV-eGFP recombinant lentivirus infection was 1.3 × 10^7^ TU/ml, and that of VSV-G/LV-RABV-G recombinant lentivirus infection was 1.1 × 10^7^ TU/ml. The titer of RABV-G/LV-eGFP recombinant lentivirus infection was 2.1 × 10^6^ TU/ml, and that of RABV-G/LV-RABV-G recombinant lentivirus infection was 1.7 × 10^6^ TU/ml. (TIF) [file pntd.0013404.s003.tif]
